# 

**Published:** 1895-04

**Authors:** 


### PERSONAL.

The practical examination at the American Veterinary College was conducted by Drs. R. S. Huidekoper, Thomas Giffen and Joseph Shea.

Dr. R. Price, of St. Paul, Minn., addressed the State Convention of Master Horseshoers on the subject of "Horseshoeing from a Scientific Standpoint."

Dr. E. H. Shepard, of Cleveland, Ohio, addressed the Master Horseshoers' Association on "The Anatomy of the Foot" at a very largely attended meeting in January last.

The following well-known veterinarians appeared before the Committee on Labor and Industries in the New York State Legislature to aid the passage of the bill to regulate the practice of horseshoeing in that State: Drs. W. H. Pendry, of Brooklyn; S. K. Johnson, of New York City, and J. C. McKenzie, of Rochester, N. Y.

We are credibly informed that with the opening of the session of 1895–96 of the New York Veterinary College that another name will be added to their teaching staff through the services of Dr. E. B. Ackerman, of Brooklyn. Dr. Ackerman is a graduate of the American Veterinary College, and is connected officially with the Brooklyn Board of Health, and has already obtained an enviable position as a successful practitioner.

Prof. R. S. Huidekoper on April 1st accepted the Chairs of Surgery and Anatomy at the New York College of Veterinary Surgeons. He will also be connected with the hospital staff and clinics, and will make his headquarters at the school. This will prove an added strength to the college in every way, and will be another step toward maintaining a higher curriculum, following its recent union with the three-year colleges of North America.

The response to the toast, "Advancement of Veterinary Science," by Prof. Rush Shippen Huidekoper at the banquet of the Alumni Association of the American Veterinary College was one of the most brilliantly interesting extemporaneous responses ever listened to. Tracing step by step its progress through ancient to modern times, naming the greater lights whose skill and renown added lustre and honor in the march of progress, not excelled, scarcely equalled by the annals of any other profession, it made one and all rejoice that they had cast their lot in the domain of comparative medicine.

The McKillip Veterinary College of Chicago will, in connection with their veterinary course, adopt one of anatomy and scientific horseshoeing for the instruction of those who are engaged in or about adopting the horseshoer's art. A similar course of instruction will also be added to the Detroit Veterinary College, and we have no doubt will prove a great benefit to the shoeing art in Michigan. We have also been informed that it is likely that the New York College of Veterinary Surgeons will establish a school of farriery in connection with their college, and that it will be equipped on a very thorough and complete basis.

We believe that schools established in conjunction with the veterinary colleges will be more likely to have a lasting and fixed existence than if started alone under the present conditions under which farriery exists in our country.

---

About 90 per cent. of all horse hides taken off are converted into leather, from which boots, shoes, gloves, and imitation buckskin are the principal products.

The annual meeting of the Veterinary Medical Association of New Jersey will be held at the State Street House, corner of State and Chancery Streets, Trenton, N. J., at 11 A.M., Thursday, April 11, 1895. A cordial invitation is extended to all members of the profession throughout New Jersey and adjoining States. W. B E. Miller, *President;* S. Lockwood, *Secretary.*

A prominent and well-known writer in an article in the *Century Magazine* for March incorrectly uses the word veterinary when he speaks of a veterinarian or veterinary surgeon. The abbreviated term "vet," frequently used in this article, is not one that is either accepted or adopted in this country. It borders too closely on slang to longer be used at all when speaking of a now recognized and acknowledged profession.

